# Learning from texts: activation of information from previous texts during reading

**DOI:** 10.1007/s11145-016-9630-3

**Published:** 2016-02-03

**Authors:** Katinka Beker, Dietsje Jolles, Robert F. Lorch, Paul van den Broek

**Affiliations:** Department of Educational Science, Leiden University, Wassenaarseweg 52, 2333 AK Leiden, The Netherlands; Department of Psychology, University of Kentucky, Lexington, KY 40506-0044 USA

**Keywords:** Intertextual integration, Multiple-text reading, Reading processes, Memory representation

## Abstract

Learning often involves integration of information from multiple texts. The aim of the current study was to determine whether relevant information from previously read texts is spontaneously activated during reading, allowing for integration between texts (experiment 1 and 2), and whether this process is related to the representation of the texts (experiment 2). In both experiments, texts with inconsistent target sentences were preceded by texts that either did or did not contain explanations that resolved the inconsistencies. In experiment 1, the reading times of the target sentences introducing inconsistencies were faster if the preceding text contained an explanation for the inconsistency than if it did not. This result demonstrates that relevant information from a prior text is spontaneously activated when the target sentence is read. In experiment 2 free recall was used to gain insight into the representation after reading. The reading time results for experiment 2 replicated the reading time results for experiment 1. However, the effects on reading times did not translate to measurable differences in text representations after reading. This research extends our knowledge about the processes involved in multiple text comprehension: Prior text information is spontaneously activated during reading, thereby enabling integration between different texts.

Learning from multiple texts is becoming increasingly important in our digitalized society. In addition to traditional paper texts, knowledge is now also delivered through websites, apps, e-mails and other new media. These different sources of information allow readers to learn about topics from multiple angles, providing texts that partially overlap and partially complement each other (Britt & Rouet, 2012; Britt, Rouet, & Braasch, 2013; Goldman, 2004; Rouet & Britt, 2011). To accomplish complete understanding of a certain topic, readers must integrate information from multiple texts. It has been argued that making connections between texts is one of the most difficult reading skills (Pearson & Hamm, 2005; Sheehan, Kostin, & Persky, 2006). However, little is known about the reading processes involved when reading multiple texts. The aim of the current study is to determine if connections between texts are created spontaneously during reading and, if so, if they affect the memory representation of the texts after reading.

## Intertextual integration during reading

Comprehension of multiple texts may involve processes that are similar to those involved in single text comprehension, including integration of new information with information stored in memory. Building on memory-based theories about single text comprehension (Albrecht & O’Brien, 1993; McKoon & Ratcliff, 1992; van den Broek, Risden, Fletcher, & Thurlow, 1996), it could be argued that information from prior texts becomes available passively without the control of the reader. Theories about single text comprehension suggest that prior information will be rapidly activated and entered into working memory through a process of resonance or spread of activation across semantic networks (Myers & O’Brien, 1998; O’Brien & Myers, 1999). This allows the construction of connections and inferences between different parts of the text and between the text and background memory (Kintsch, 1988; McNamara & Magliano, 2009; van den Broek et al., 1996). Activation of prior information depends on featural overlap between current and prior information—including overlap of protagonist, action, or context—and results in faster processing of the new information (Dell, McKoon, & Ratcliff, 1983; Duffy & Rayner, 1990; O’Brien, Duffy, & Myers, 1986). For example, it has been demonstrated that it takes less time to resolve an anaphor that shares several characteristics with an antecedent than an anaphor that shares only a few characteristics with an antecedent (e.g., when the anaphor is a synonym of the antecedent) (Dell et al., 1983; McKoon & Ratcliff, 1980).

There might also be differences in the processing of single and multiple texts. For example, featural overlap across multiple texts may be reduced due to differences in superficial characteristics related to the context in which the information was read (e.g., when two texts are read in a different location, time, or modality) and the source of information (e.g., the person or organization providing the information). When featural overlap is low, prior text information may not be activated during reading a subsequent text, and connections between texts may not be created. Previous research has demonstrated that abstract knowledge (i.e. a schema) from previous texts is activated directly after reading subsequent texts, but only with strategic effort (Seifert, McKoon, Abelson, & Ratcliff, 1986). In their study each text could be comprehended independently from the other texts. However, often comprehension of a text requires comprehension of specific content in another text, especially when learning about complex topics. Therefore, the first experiment in the current study was designed to gain more insight into on-line processes during reading multiple texts in situations where prior texts are required for comprehending subsequent texts. More specifically, we wanted to determine whether concurrent activation of information from multiple texts occurs during reading. Because concurrent activation is argued to be a precondition of integration within single texts (Kendeou & O’Brien, 2014), we anticipate that it will be important for integration across multiple texts.

## Intertextual integration in memory representations after reading

With respect to the representation of text information in memory, comprehension of multiple texts is successful when readers construct a representation that integrates the most important information from different texts to create a coherent whole (Britt, Perfetti, Sandak, & Rouet, 1999). Text representations can be visualized as networks with nodes representing concepts from the texts and background memory, and links representing connections between the concepts. A representation of multiple texts requires connections between information units from different parts of a single text (intratextual connections) and connections between information units from different texts (intertextual connections). During reading, each subsequent text may change, strengthen or add nodes and links to the existing memory representation. However, it does not necessarily follow that different texts are integrated into one shared memory representation.

There are several factors that influence whether multiple texts are integrated in memory. One important factor is conceptual consistency between the texts. If information from different texts is inconsistent, it is difficult to integrate the information into a single representational network. Readers could cope with this by tagging the inconsistent information to different sources in memory or by qualifying connections with labels such as ‘is inconsistent with’ (Britt et al., 1999).

Another factor that influences intertextual integration in memory is the context in which the texts are presented (e.g., the physical, temporal, and functional context) and the source of the information. The larger the distance between the contexts in which the texts are read, the more difficult it will be to integrate the information in memory because it may be less obvious that the texts are related. This may result in compartmentalization of the representation, showing mainly intratextual connections and fewer intertextual connections. Even when the distance in reading contexts is small, a perceptual or semantic boundary may be sufficient to elicit distinct reading processes that hinder intertextual integration. For example, research has shown that different processes occur at the beginning and the end of a text (Gernsbacher, 1990). It has been argued that the beginning of a text functions as a foundation to which new information is mapped (Gernsbacher, 1990). With every new text, this process may start anew (Britt et al., 2013). Moreover, wrap-up effects have been perceived at constituent boundaries (Just, Carpenter, & Woolley, 1982), such as at the end of clauses (Aaronson & Scarborough, 1976), sentences (Rayner, Kambe, & Duffy, 2000), and arguably texts as well. These processes may contribute to compartmentalization of the representation of different texts, making it more difficult to create intertextual connections.

Previous research has demonstrated that multiple texts are integrated in memory when they share a text structure (McKoon, Ratcliff, & Seifert, 1989; Seifert et al., 1986), but this process is dependent on instructions when content overlap is low (Seifert et al., 1986). In addition, memory for a subsequent text is facilitated when it is preceded by a text that has a similar text structure (Thorndyke, 1977). In these studies the paired texts were causally unrelated. When multiple texts are causally related, such as in the current research, building an accurate text representation requires integration of information from both texts.

In summary, multiple factors are likely to influence whether a reader integrates related information across distinct texts. The first experiment adapts the contradiction paradigm (Albrecht & O’Brien, 1993) to investigate whether readers activate relevant information from a previous text when they read a target sentence. The materials of the first experiment were intentionally designed to favor such activation by presenting short, related texts consecutively and using pairs of context and target sentences with high featural overlap. The second experiment was designed to gain insight into how multiple text processes during reading relate to the resulting memory representation in situations where prior texts are required for comprehending subsequent texts. Specifically, we wanted to know whether readers are more likely to include intertextual connections in memory in situations in which intertextual connections help to restore comprehension and whether the processing time of information during reading multiple texts is related to the prominence of that information in memory.

## Experiment 1

The goal of the first experiment in the current study was to examine whether readers spontaneously activate information from a previously read text during reading when it is relevant to understanding the text they are currently reading. To test this, we created a multiple-text integration paradigm based on the contradiction paradigm (Albrecht & O’Brien, 1993). Using the contradiction paradigm, it has been demonstrated that information is processed more slowly when it is preceded by inconsistent information than when it is preceded by consistent information. This shows that prior information from the same text is activated during reading of subsequent sentences. In the multiple-text integration paradigm we also included consistent texts (Consistent condition) and texts with inconsistencies. The texts with inconsistencies were preceded by separate texts that either contained information that could be used to restore coherence in the subsequent text by explaining the inconsistency (Inconsistent-with-explanation condition), or by texts that contained neutral information that could *not* be used to restore coherence in the subsequent text (Inconsistent-without-explanation condition). If information from the first text is available during reading of the second text, then the activation of explanatory information should facilitate processing of the second text because the explanation restores coherence of the text. If the first text does not provide an explanation, coherence cannot be restored and processing will not be facilitated. Consider reading “A rulver is brown. It is difficult to see in the white snow”. The second sentence, in which the inconsistency unfolds, presumably requires a longer time to process compared to the same phrase in the Consistent condition, “A rulver is white. It is difficult to see in the white snow.”, because the information is difficult to integrate with prior knowledge. However, coherence could be restored by activating information from a previous text that stated that “In the winter, the rulver’s fur changes to white”. With this information you can infer that rulvers are brown in the summer and that they become white in the winter, which makes them difficult to see in the white snow. Reading times are expected to be faster in this case. If the previous text does not provide an explanation, then the inconsistency in the second text remains unresolved and reading times are not expected to speed up.

## Method

### Participants

Participants were 27 Leiden University undergraduates studying education sciences or psychology. Participants’ ages ranged between 18 and 32 with a mean of 19.2 years (2.3 SD). All participants were female except one, and all were fluent Dutch speakers. All participants had good or corrected eyesight and lacked reading problems or learning disabilities. Students could submit to participate in the study by signing up at the Leiden University Research Participation system. Informed consent was obtained for all participants. Participation was rewarded with course credits.

### Materials and design

Example materials are presented in Table 1. The texts described 30 topics in expository text format.^1^ The texts were short in length (with an average number of 5.5 sentences) and described information about animals, persons, objects, countries, and events. Fictitious topics were used to equate prior knowledge, by replacing the names of real-world topics by fictitious ones (e.g., the text about the ‘rulver’ was based on the polar fox). For each topic there were three versions of the text/text pair, which were counterbalanced across subjects: Consistent texts; inconsistent texts in combination with preceding texts containing an explanation; and inconsistent texts in combination with preceding texts omitting an explanation.

**Table 1:** Example text materials showing three versions of the topic ‘The Rulver’

Inconsistent-with-explanation	Inconsistent-without-explanation	Consistent
*Text 1*		
The rulver is an animal that lives on heathland. It has a pretty brown fur for which hunters can get a lot of money. But in the winter they stop hunting the rulver. *In the winter, the color of the rulver’s fur changes to white*.	The rulver is an animal that lives on heathland. It has a pretty brown fur for which hunters can get a lot of money. But in the winter they stop hunting the rulver. *The hunters have to get their money from another source to be able to get enough income*.	–
*Text 2*		
The rulver’s fur has a beautiful brown color and is therefore very popular. Many hunters search for rulvers. But in the winter they stop hunting the rulver. It is not easy to spot the rulver in the *white* snow. The hunters have to wait until the snow disappears.	The rulver’s fur has a beautiful brown color and is therefore very popular. Many hunters search for rulvers. But in the winter they stop hunting the rulver. It is not easy to spot the rulver in the *white* snow. The hunters have to wait until the snow disappears.	The rulver’s fur has a beautiful white color and is therefore very popular. Many hunters search for rulvers. But in the winter they stop hunting the rulver. It is not easy to spot the rulver in the *white * snow. The hunters have to wait until the snow disappears.

The differences between first texts in the Inconsistent-with-explanation and Inconsistent-without-explanation condition are italicized. The underlined word is what makes the underlined target sentence inconsistent (in the Inconsistent-with-explanation and Inconsistent-without-explanation conditions) or consistent (in the Consistent condition). These sample texts are translated from Dutch

Thus, the Inconsistent-with-explanation condition consisted of two texts. The first text contained an explanation for an inconsistent target sentence in the second text. The target sentence in the second text was always the penultimate sentence of the text, and the information in this sentence was inconsistent with the information that preceded the target sentence in the same text.

The Inconsistent-without-explanation condition also consisted of two texts, but in this condition the first text did not contain an explanation for the inconsistent target sentence in the second text. Instead, the first text described additional information about the topic.

The Consistent condition consisted of only one text. This text was similar to the second text in the Inconsistent conditions, with the exception that the information that preceded the target sentence was consistent with the target sentence. The target sentences were exactly the same in the three versions of each topic, but differed between different topics. The target sentences had an average length of 61 (SD = 19) characters.

### Procedure

Each testing session lasted about an hour. Participants first received verbal instructions about the procedure of the experiment on the computer. They were told that they were going to read texts sentence-by-sentence and they were asked to read these texts for comprehension and to answer questions about these texts. The questions were included to determine whether the participants were paying attention.

After the verbal instructions, participants were asked to read the same instructions on the screen, and they performed one practice trial. The experimenter gave feedback during the practice trial if necessary. If participants demonstrated comprehension of the task during the practice trial, they were instructed to continue through the remainder of the experiment individually and feedback was no longer provided.

Before each text was presented, “NEXT TEXT” was presented in the center of the display screen to indicate the beginning of a new text. The next screen showed a fixation cross in the center of the screen that was presented for a variable interval of between 500 and 2500 ms. Sentences were presented one by one. Participants were instructed to read at their own pace. They could progress to the next sentence by pressing the space bar. To prohibit readers from skipping a sentence by accidentally double-hitting the space bar, the program did not respond to a press if it occurred within 500 ms of the previous press. Also, if readers took longer than 10.000 ms to read a sentence the program automatically continued to the next sentence. After reading each text, participants were presented with a question about a section of the text; the question was the same in all conditions. The questions could be answered with yes or no. The participants were instructed to keep their thumbs on the space bar, and their index fingers on the “yes” and “no” keys at all times (the “S” and “L” keys on the keyboard). They did not receive feedback about the accuracy of their answers. The topics and conditions were presented in different orders for each participant. Half of the participants received the topics in one order, the other half in the reversed order. The order of conditions was counterbalanced by a Latin square procedure. Texts that were related (in the inconsistent conditions with and without explanations) were always presented in a consecutive order but, like all texts, were separated by a comprehension question and the message “NEXT TEXT”.

### Recording data

Reading times between onset of presentation of each sentence and the press of the space bar were recorded. The analyses involved the reading times of the target sentences and the sentences that followed the target sentences (the latter to investigate spillover effects).

## Results

Before analyzing the data, the responses to the questions and the reading times were inspected. On average, participants answered 89 % of the questions correctly, which shows they were paying attention to the texts. Reading times that deviated over 2.5 standard deviations on both the subject and item means were removed, assuming these were situations in which participants were not following the task instructions (for example, because they were distracted). Less than 1 % of the data were removed using this criterion. The descriptives are displayed in Table 2.

**Table 2:** Mean reading times (in ms) and standard deviations (in parentheses) for the target sentences for each condition in experiment 1

Condition	Mean (SD)
Inconsistent-with-explanation	2685.23 (1252.34)
Inconsistent-without-explanation	2904.04 (1388.90)
Consistent	2618.84 (1321.11)

As the distribution of the reading times was skewed to the right, the reading times were transformed by taking the natural log of each score to make the distribution more symmetrical (Richter, 2006). Because of the multilevel structure of the data (Richter, 2006), reading times were analyzed using hierarchical linear models using R-statistics software and the LmerTest package. Item-level reading speeds were clusters at Level 1 and subjects and items were clusters at Level 2, with the items nested within conditions. Subjects and items were treated as random effects whereas the conditions were treated as a fixed factor with three levels.^2^ Degrees of freedom are estimated with Satterthwaite’s approximation method (Kuznetsova, Brockhoff, & Christensen, 2015; SAS Technical Report, 1978; Satterthwaite, 1941). Effects will be classified as significant when *p* < .05. Restricted maximum likelihood was used to fit the models. First a baseline model was fit with random intercepts for subjects and items, and this model was compared to a model that also included the conditions.

The results show that adding the conditions made a significant contribution to the model compared to a baseline model [χ^2^(2) = 12.59, *p* = .002]. In agreement with previous research, the mean reading time of the target sentence in the Inconsistent-without-explanation condition was significantly slower than the mean reading time of the target sentence in the Consistent condition [*b* = .10, *SE* = .03, *t*(748) = 3.46, *p* < .001]. In addition, the mean reading time of the target sentence in the Inconsistent-with-explanation condition was significantly faster than the mean reading time of the target sentence in the Inconsistent-without-explanation condition [*b* = .07, *SE* = .03, *t*(743) = 2.43, *p* = .016]. There were no significant differences in average reading times of the target sentence in the Inconsistent-with-explanation and the Consistent condition [*b* = .03, *SE* = .03, *t*(748) = 1.07, *p* = .29]. The sentence that followed the target sentence was also analyzed, but the conditions did not significantly contribute to the model compared to a baseline model, indicating that there were no spill-over effects [χ^2^(2) = 2.38, *p* = .304].

### Summary of results experiment 1

The results of experiment 1 demonstrate that prior texts with explanations facilitated processing of inconsistent information in the subsequent texts. This shows that information from prior texts is activated during reading. The reading speed in the Inconsistent-with-explanation condition was more similar to the Consistent condition than the Inconsistent-without-explanation condition, suggesting that activation of the information from prior texts helped to restore coherence. The results are in accordance with the notion that memory-based processes extend beyond textual boundaries: The inconsistent information in the second text seems to have passively activated the explanation from the first text. This experiment is the first to show that intertextual integration (i.e. activation of prior texts) takes place during reading.

## Experiment 2

### Intertextual integration and prominence of information in memory

In the single text research it has been repeatedly demonstrated that reading processes influence the memory representation of the texts (van den Broek et al., 1996). One purpose of experiment 2 was therefore to determine whether intertextual connections are included in the memory representation of the texts. This was done by asking readers to recall what they remembered from the texts after having read several other texts in between. Two aspects of the memory representation were investigated: (1) intertextual integration, and (2) inclusion of different types of information.

Experiment 1 provided evidence for the activation of prior text information during reading a second text. This means that information from two texts was active at the same time and this is a necessary precondition for intertextual integration (Kendeou & O’Brien, 2014; Kendeou, Walsh, Smith, & O’Brien, 2014; van den Broek & Kendeou, 2008). If co-activation of the two texts indeed led to intertextual integration during reading, it is likely that these connections will also be included in the memory representation. Intertextual integration in memory was assessed by determining whether readers report unique information from both texts in one recall session.

Memory is often better for inconsistent information because it is more salient (e.g., Rojahn & Pettigrew, 1992; Sakamoto & Love, 2004; Stangor & McMillan, 1992). It could be argued that the inconsistency is more salient in the Inconsistent-without-explanation condition than in the Inconsistent-with-explanation, because in this condition it cannot be resolved with information from the text. Therefore, it can be expected that the inconsistency is more prominent in memory. Furthermore, previous research has shown that information that is activated more often or longer during reading is more prominent in the memory representation (van den Broek et al., 1996). This would also lead to the expectation that the inconsistency is more prominent in the memory representation in the Inconsistent-without-explanation condition than in the Inconsistent-with-explanation condition, because the target sentence was read slower. Alternatively, because readers strive for coherence, readers may choose to ignore information that does not fit the representation (Maier & Richter, 2013; Stadtler, Scharrer, & Bromme, 2012). The inconsistent information may therefore be less prominent in memory in the Inconsistent-without-explanation condition than in the Inconsistent-with-explanation condition. To examine the prominence of the inconsistency in the memory representation, we determined whether readers recalled the target and/or context information, which both make up the inconsistency.

### Elaboration

To ensure that observed differences between conditions are based on differences in semantic representation in memory rather than in superficial memory traces, recall was administered after a delay. Such a delay carries the potential risk that information would decay from the memory representation, thereby decreasing the chance of observing differences in representation between the conditions. Therefore, in experiment 2 the central information in the initial texts was expanded by elaborating on the explanation (in the Inconsistent-with-explanation condition) and on neutral information (in the Inconsistent-without-explanation condition, to match the text length). Previous research has shown that elaborated information results in richer memory representations than unelaborated information (Bradshaw & Anderson, 1982) and this improves activation of elaborated information at a later moment in time because of the multiple retrieval routes. To allow for comparisons with experiment 1, and to leave open the possibility that elaboration interacts with the experimental conditions, elaboration was included as an additional factor: Elaboration and explanation were combined in a 2 × 2 design with four inconsistent conditions formed by crossing (1) the presence versus absence of an explanation, and (2) the presence versus absence of elaboration).

## Method

### Participants

Participants were 32 Leiden University undergraduates studying education sciences or psychology. Participants’ ages ranged between 18 and 28 with a mean of 20.7 years (2.2 *SD*). Of all participants, 26 were female and 6 were male, and all were fluent Dutch speakers. All participants had good or corrected eyesight and lacked reading problems or learning disabilities. Students could submit to participate in the study by signing up at the Leiden University Research Participation system. Informed consent was obtained for all participants. Participation was rewarded with course credits or gift cards (whatever they preferred).

### Materials and design

The design and materials of experiment 2 were based on experiment 1 but slight changes were made to fit the purposes of experiment 2. First, to examine the effects of elaboration on reading times and recall, experiment 2 included two additional inconsistent conditions in which the first texts were extended with three to five sentences. In the elaborated Inconsistent-with-explanation condition, the additional sentences expanded the section of the context text that provided the explanation for the target sentence. For example, in the text about the rulver the explanation is elaborated by describing the mechanisms (sunlight, melanin) that cause the change in color of its fur. In the Inconsistent-without-explanation condition, the added information was irrelevant to the target sentence. The Consistent condition was not included in experiment 2. All other text characteristics were kept as similar as possible. Taken together, experiment 2 included four inconsistent conditions formed by crossing two factors: (1) presence versus absence of an explanation, and (2) presence versus absence of elaboration. As in experiment 1, the reading times of the target sentences and the sentences that followed the target sentences were recorded.

Second, participants in experiment 2 were asked to recall what they remembered from each text after reading four text pairs. Participants were asked to report the most important information they remembered from the text. The questions always followed the same format: “What do you remember from the text about topic X?”, where X represents the main topic of the two texts (often the fictitious animal/object/person, for example the ‘rulver’). Participants were asked to type their answers on the computer. Next, a question was asked about the target sentence. For example, the target sentence “It is difficult to see in the white snow” would be queried by “Why is it difficult to see the rulver in the white snow?”. The right answer to this question involves the explanation (“It’s fur turns white in the winter”). The purpose of this question was to check whether the manipulation of elaboration on the explanation was effective. If elaboration prevents the decay of important information from memory, than recall of the explanation should be higher in the elaborated conditions compared to the unelaborated conditions.

### Procedure

The procedure was the same as in experiment 1 with the exceptions that participants had to recall information from the texts and answer questions about the texts after reading four text pairs. In addition, the text-based questions from experiment 1 were omitted to save time. The memory questions were presented in the same order as the participants read the texts. Due to the addition of the memory questions, the testing session lasted on average half an hour longer than in experiment 1. Four participants did not complete the entire test because of time limitations.

### Scoring free recall

All variables were scored dichotomously (yes/no). To assess integration we used a liberal criterion: Integration was scored positively when participants mentioned unique information from both the first and the subsequent text. Integration was scored negatively when participants reported information from only the first or the second text, or from neither text. To assess recall of the inconsistency, two variables were created. One variable indicated whether readers mentioned information from the target sentence, and one indicated whether readers mentioned the context information with which the target sentence is inconsistent. The scoring was done by the first author and a trained research assistant. The inter-rater reliability was high (.85 ≤ κ ≤ .95). Disagreements were resolved by discussions.

## Results

### Reading times

The same selection criterion as in experiment 1 was used to remove outliers (less than 1 % of the data were deleted). The descriptives are displayed in Table 3.

**Table 3:** Mean reading times (in ms) and standard deviations (in parentheses) for the target sentences for each condition in experiment 2

	With-elaboration	Without-elaboration
Inconsistent-with-explanation	2104.93 (1127.05)	2130.07 (1046.38)
Inconsistent-without-explanation	2313.91 (1180.54)	2394.24 (1347.69)

The data were analyzed analogously to experiment 1. The results show that adding the two factors Explanation (with or without) and Elaboration (with or without) together made a significant contribution to the model [χ^2^(3) = 16.85, *p* < .001]. However, only the factor of Explanation made a significant contribution to the model [*b* = .09, *SE* = .03, *t*(1008) = −2.90, *p* = .004]. The mean reading time of the target sentence in the Inconsistent-with-explanation condition was significantly faster than the Inconsistent-without-explanation condition, replicating the results of experiment 1. Elaboration did not make a significant contribution [*b* = .02, *SE* = .03, *t*(1008) = .67, *p* = .501] nor did the interaction between Explanation and Elaboration [*b* = −.01, *SE* = .05, *t*(1008) = −.13, *p* = .898].

To determine spill-over effects, the same analysis was repeated with the reading times on the sentence that followed the target sentence as dependent measure. The results were analogous to the results on the target sentence: Inconsistent-with-explanation texts were read faster than Inconsistent-without-explanation texts [*b* = .09, *SE* = .03, *t*(1004) = 3.22, *p* = .001]. The other effects were not significant.

### Free recall

Free recall was analyzed using the same procedures as in the previous analyses with the exception that now *logistic* hierarchical linear models was applied with Maximum Likelihood to fit the models. Table 4 provides an overview of the mean proportions of the recall measures for each condition. Neither factor influenced any of the recall measures: Comparison of the model with both factors included to the baseline model omitting the two factors did not approach significance for the integration measure [*χ*^2^(3) = 4.45, *p* = .22], or for the recall of the inconsistency; consisting of the context information [χ^2^(3) = 3.69, *p* = .30] and the target information [χ^2^(3) = 3.79, *p* = .28].

**Table 4:** Proportion of integration in recall reports and recall of context and target information for each condition in experiment 2

Explanation	Elaboration	Mean integration (SD)	Mean target (SD)	Mean context (SD)
Yes	Yes	.82 (.39)	.43 (.50)	.57 (.50)
Yes	No	.75 (.43)	.47 (.50)	.62 (.49)
No	Yes	.79 (.40)	.43 (.50)	.59 (.49)
No	No	.78 (.42)	.49 (.50)	.54 (.50)

### Manipulation check

With regard to the results on the specific question that cued the explanation, the model with the factor Elaboration included had a better fit compared to the baseline model [χ^2^(1) = 12.69, *p* < .001]. Recall of the explanation was higher in the elaborated condition (*M* = .75, SD = .43) compared to the unelaborated condition (*M* = .63, SD = .48), (*b*_*elaboration*_ = .75, *SE* = .21, z = 3.56, *p* < .001). This finding shows that the manipulation of elaboration was successful.

### Summary of results experiment 2

The results of experiment 2 demonstrate that prior texts with explanations facilitated processing of inconsistent information in the subsequent texts. This replicates experiment 1 and provides converging evidence that information from prior texts is activated during reading and that activation of prior text information facilitates the reading process (as reflected by faster reading times). Experiment 2 did not find evidence for a relation between the reading processes and the resulting memory representation. Differences in the activation of information during reading were not reflected in differences in intertextual integration and prominence of information in memory (i.e. the inconsistency).

## Discussion

Learning from texts often involves the integration of information from multiple texts. Intertextual integration requires the activation of information from a prior text during reading of a subsequent text. The goal of the present study was to determine whether information from a previously read text is spontaneously activated during reading of a novel text and whether this affects the representation of the texts. The results of the first experiment show that the processing of inconsistent information was faster when a prior text contained an explanation for the inconsistency. In the second experiment, memory of the texts after a delay was assessed in addition to the reading processes. The reading processes showed a similar pattern as in experiment 1. Two aspects of memory were investigated: Intertextual connections and prominence of information (i.e. the inconsistency) in memory. Results indicate that the processing differences did not affect the presence of intertextual connections that were encoded in memory, nor did it influence the prominence of the inconsistent information in memory.

### Intertextual integration during reading

The results of both experiments show that prior texts with explanations speed up processing of inconsistencies in a subsequent text. This suggests that activation of the explanations from previously read texts facilitated the resolution of the inconsistent information during reading, resulting in more coherence and, consequently, in faster reading. Results from prior research have demonstrated facilitative effects of background knowledge on text comprehension (Elbro & Buch-Iversen, 2013; McNamara & Kintsch, 1996; McNamara, Kintsch, Songer, & Kintsch, 1996). The current study extends these findings by showing that recently read texts about the same topic also facilitate comprehension of subsequent texts.

Because participants in the current study did not receive instructions to integrate information across texts, it is likely that the explanations were activated spontaneously. This is in line with memory-based theories of information processing developed in the context of single-text processing (Albrecht & O’Brien, 1993; McKoon & Ratcliff, 1992; van den Broek et al., 1996). As in the context of single texts, spontaneous activation of prior text information may have been triggered by featural overlap between the preceding and subsequent text (Albrecht & Myers, 1998; Albrecht & O’Brien, 1993; O’Brien & Albrecht, 1991), for example because they were about the same topic. This featural overlap may have led to co-activation of the prior and current text information and, consequently, to intertextual integration (Kendeou & O’Brien, 2014).

Activation of prior information has been shown to spread from recently read and more central information in memory to more distant and less central information in a backward parallel search (O’Brien, 1987; O’Brien, Plewes, & Albrecht, 1990). In the condition with explanation, the explanation may have been quickly activated during a backward parallel search because the previous text was read recently and had a high featural overlap with the current text. In the conditions without explanation, there was no explanation to be activated during a backward parallel search. The failure of the activation process to locate any connections that might resolve the inconsistency may have led to an extended search process that took more time, explaining the relatively long reading times on target sentences in the conditions without explanations.

Although not central to the purposes of the study, it is interesting to note that the results of experiment 2 show that elaboration of information in the first text did not influence the processing speed of the target information in the second text. This is not surprising, given that the activation of prior text information was already optimal in the condition with explanations and without elaboration (i.e. the processing speed was the same as when reading consistent information). It is possible, however, that elaboration does facilitate activation of prior text information in more challenging situations. Additional research is necessary to draw reliable conclusions about the influence of elaboration on activation of prior text information.

### Intertextual integration in memory representations after reading

The second experiment was designed to investigate the relation between intertextual reading processes and the resulting memory representation. Free recall was used to assess memory for intertextual connections and prominence of the inconsistency. There were no significant differences between the conditions on either measure. This seems inconsistent with previous findings that reading processes correlate with memory (Tzeng, van den Broek, Kendeou, & Lee, 2005; van den Broek et al., 1996). One possible explanation for the null effects is that relatively small differences in processing during reading are not sufficient to produce more permanent effects on memory. However, it is also possible that there *are* effects on memory but that our recall measures are not sufficiently sensitive to capture the effects. For example, the measure of intertextual connections in memory was a dichotomous measure that may have been too gross to reveal differences in integration between the conditions. Relatedly, recall measures require respondents to make a decision about what to report and this may not accurately reflect the actual memory representation (McKoon & Ratcliff, 2015). Other measures, such as priming, might be more effective in demonstrating effects of reading processes on memory.

### Limitations

In this study the distance between the two related texts was small, raising the question whether readers perceived the texts as two distinct entities. However, several text and context cues were provided to create a distinctive boundary between the texts (Britt et al., 2013). In experiment 1 there were three cues for the readers that indicated the end of the previous text and the beginning of a new text. (1) The intervening task: Every text was followed by a comprehension question about the previous text. (2) The heading: Before each new text the message “NEXT TEXT” occurred on the screen. This cue demarcates a distinct section because it instructs the reader to build a new mental representation, and it is typographically different from the text sentences (Lorch, 1989). (3) The structure: The texts were designed to be independent and they can be comprehended individually (with the exception of the inconsistency) because of their syntactic structure. Every text began with an introductory sentence, ended with closing sentence, and each concept was introduced as if it were new. In experiment 2 these cues were the same with the exception of the first cue. These cues indicate readers that they are reading multiple texts and not just paragraphs of a single text.

Although we believe that readers perceived related texts as distinct texts, we view the experiments as the initial step in investigating the integration of information across texts. The conditions under which such integration was investigated in this study represent minimal challenges to readers’ abilities to integrate information. However, it is necessary to first establish that readers can accomplish integration under favorable conditions before attempting to determine the boundary conditions for such integration. Clearly, it is important to extend the current research to reading situations that are more authentic. Future research with the multiple-text integration paradigm should include texts that more closely align complex, realistic situations. A reasonable first step would be to increase the textual or physical distance between the texts to determine which factors decrease intertextual integration in more difficult situations. In addition, as with integration within a single text, intertextual integration may be affected by text factors such as featural overlap and strategies, and individual differences such as working memory, background knowledge etc. Finally, to obtain a better indication of the characteristics of the memory representation, different measures could be used, including more implicit measures such as priming, which minimize post-reading strategic processes (McKoon & Ratcliff, 2015).

In conclusion, it is common to encounter different treatments of the same topics in different sources. To form an integrated perspective on a complex topic, readers must (at least implicitly) recognize when something they are currently reading overlaps with knowledge they have gained from another source. Such recognition is the first step to integrating related information from multiple sources. The multiple text integration paradigm introduced in this study is a first step towards understanding the processes underlying the integration of information across multiple sources.

## Footnotes

1 Expository texts were used because it is common to use this genre to present new ideas (Singer, 2015).

2 The data were also analyzed with sentence length (number of characters) as additional fixed factor, but because the results were the same, only the model without sentence length is described.
